# Genome sequences of *bla*_NDM-1_ producing *Acinetobacter lactucae* isolated from immunocompromised patients in India

**DOI:** 10.1128/MRA.00220-23

**Published:** 2023-10-11

**Authors:** Ashutosh Kumar Amar, Ajit Ramesh Sawant, Meerabai Manoharan, Archana Tomar, Sujatha Sistla, K Prashanth

**Affiliations:** 1 Department of Biotechnology, School of Life Sciences, Pondicherry University, Pondicherry, India; 2 Department of Microbiology, Jawaharlal Institute of Postgraduate Medical Education and Research (JIPMER), Pondicherry, India; 3 Helmholtz Centre Munich, German Research Center for Health and the Environment (GmbH), Institute of Experimental Genetics, Ingolstadt Landstrasse, Neuherberg, Neuherberg, Germany; University of Maryland School of Medicine, Baltimore, Maryland, USA

**Keywords:** genome analysis, *Acinetobacter lactucae*, NDM-1

## Abstract

Among the species within *Acb-*complex, *Acinetobacter lactucae* has not been frequently isolated from clinical settings, unlike *Acinetobacter baumannii*, which is an important nosocomial pathogen. We report the genomic sequences of *A. lactucae* strains (PKAL1732 and 1828C) harboring multiple-resistance determinants including metallo-β-lactamase (*bla*_NDM-1_) isolated from immunocompromised patients admitted to a referral hospital in India.

## ANNOUNCEMENT

The emergence of non-baumannii *Acinetobacter* in hospitals worldwide highlights the importance of other species of the genus *Acinetobacter* in a changing healthcare scenario (1 – 3). With the increased isolations of resistant non-baumannii *Acinetobacter*, whole-genome sequencing (WGS) may help investigating the nature of their infection, resistance, and pathogenicity. Here, we present the WGS of two *Acinetobacter lactucae* isolates, which were isolated from immunocompromised patients hospitalized in an Indian hospital. The isolates that were analyzed were designated as PKAL1828C and PKAL1732, wherein the first isolate was obtained from blood culture, and the second isolate grew on a blood agar plate from endotracheal aspirate specimen after overnight incubation at 37°C with 5% CO_2_. The 16S rRNA gene sequences used for the identification of *A. lactucae* as described elsewhere (4) have been deposited in GenBank under accession numbers MN861122 and MN861123. WGS analysis of *A. lactucae* may help in understanding the nature of this pathogen and its pathogenic potential.

*A. lactucae* genomic DNA was isolated using the HiPurA DNA Isolation Kit (Hi-Media, India) from a 5-mL overnight culture grown at 37°C in Luria broth. DNA sequencing was performed using Illumina platform, where paired-end library was constructed using Nextera XT DNA Library Preparation Kit (Thermo Scientific, USA), and the library quality was checked with Agilent Technologies 2,100 Bioanalyzer using a DNA 1000 chip. Subsequently, the paired-end sequence reads were trimmed to remove adapter sequences and low-quality reads with Phred quality scores <20 using cutadapt (v 3.5). The filtered paired-end reads were *de novo* assembled using SPAdes v3.15.3. with default parameters (5). The final assembled genome size is 4,266,510 bp for PKAL1732 and 4,168,179 bp for PKAL1828C with a GC content of ~39%. The contigs obtained from *de novo* assembly were reordered against genome sequences of reference genome *A. lactucae* ANC4052 (NZ_KB976991) using Mauve Contig Mover (6). Genome annotation was performed using the NCBI Prokaryotic Genome Annotation Pipeline version 6.4 (7). Resistance-related genes and insertion sequences were analyzed using ResFinder (version 4.1) (8) and mobile genetic elements (MGE v1.0.3), respectively (9). Default parameters were used for all software unless otherwise specified. All the genome features of *A. lactucae* strains were given in Table 1. This study reports the presence of *bla*_NDM-1_ in *A. lactucae* strains, which were tested for their susceptibility by determining the minimum inhibitory concentrations (MICs) for nine antibiotics by agar dilution method following the norms of the Clinical Laboratory Standards Institute (CLSI) (10). The susceptibility results confirmed *A. lactucae* strains were multiple drug resistance (MDR) phenotype as they were resistant to at least three different classes of antimicrobial agents (Table 1). Identification of different β-lactamase genes including *bla*_NDM-1,_ macrolide resistance genes such as *msr(E*) and *mph(E*), and aminoglycoside modifying enzyme *aph(3')-VIa* might be responsible for MDR phenotype. Moreover, an MGE finder identifies unique insertion sequences in each genome except for IS*Aba*125, which is common in both strains. The presence of resistance genes, genes encoding biofilm-associated proteins, and type-VI secretion system indicates the possible pathogenic potential of *A. lactucae*; however, the functions of these genes are yet to be experimentally confirmed.

**TABLE 1:** Genetic features of *Acinetobacter lactucae* isolates PKAL1732 and PKAL1828C

Feature	PKAL1732	PKAL1828C
Site of isolation	Endotracheal aspirate	Blood
Annotation Pipeline Version 6.4	PGAP^*b*^	PGAP
No. of reads	14.32 M	14.32 M
Genome size (bp)	4,266,510	4,168,179
Contigs	81	340
Coarse consistency	99.4	99.5
Fine consistency	98.3	98.6
GC content (%)	38.84	38.97
L50	4	15
N50 (bp)	355,961	98,221
Genes (total)	4,112	4,057
CDSs (total)	4,053	3,987
Genes (coding)	3,989	3,883
CDSs^*a*^ (with protein)	3,989	3,883
rRNAs (5S, 16S, and 23S)	1, 1, and 1	3, 1, and 3
tRNAs	52	59
Genome coverage	100×	99×
Susceptibility results		
Antibiotic (MIC mg/L)		
Tobramycin	0.62	0.50
Amikacin	256	>1,024
Meropenem	>256	0.75
Ciprofloxacin	512	512
Ceftazidime	>256	14
Gentamicin	256	8
Ceftriaxone	256	16
Tigecycline	0.50	0.125
Colistin	0.75	0.50
Genetic resistance determinants		
Sulfonamide resistance	-	*sul2*
Tetracycline resistance	*tet(39*)	
β-lactam resistance	*bla_NDM-1_, bla_OXA-270_, bla_ADC-25_*	*bla_OXA-270_ bla_ADC-25_*
Aminoglycoside resistance	*aph(3')-VIa*	
Macrolide resistance	*msr(E), mph(E*)	*msr(E), mph(E*)
Database accession no.		
GenBank no.	GCA_028774625.1	GCA_028774645.1
BioProject no.	PRJNA817733	PRJNA817733
BioSample no.	SAMN26806653	SAMN26806654
SRA accession number	SRX19739304	SRX19739305

^*a*^ CDSs, coding DNA sequences.

^*b*^ PGAP, Prokaryotic Genome Annotation Pipeline.

## Data Availability

This whole-genome project has been deposited in DDBJ/EMBL/GenBank under accession numbers JALNTF000000000 and JALNTG000000000 for *A. lactucae* PKAL1732 and *A. lactucae* PKAL1828C, respectively, as well as BioProject number PRJNA817733 and BioSample numbers SAMN26806653 and SAMN26806654.
